# Exercise-Induced Myokines can Explain the Importance of Physical Activity in the Elderly: An Overview

**DOI:** 10.3390/healthcare8040378

**Published:** 2020-10-01

**Authors:** Jenny Hyosun Kwon, Kyoung Min Moon, Kyueng-Whan Min

**Affiliations:** 1 Department of Kinesiology, Indiana University, Bloomington, IN 47405, USA; jenkwon@iu.edu; 2 Department of Pulmonary, Allergy, and Critical Care Medicine, GangNeung Asan Hospital, University of Ulsan College of Medicine, Gangneung 25440, Korea; 3 Department of Pathology, Hanyang University Guri Hospital, Hanyang University College of Medicine, Guri 11923, Korea; kyueng@gmail.com

**Keywords:** myokine, elderly, exercise, physical activity, aerobic, anaerobic

## Abstract

Physical activity has been found to aid the maintenance of health in the elderly. Exercise-induced skeletal muscle contractions lead to the production and secretion of many small proteins and proteoglycan peptides called myokines. Thus, studies on myokines are necessary for ensuring the maintenance of skeletal muscle health in the elderly. This review summarizes 13 myokines regulated by physical activity that are affected by aging and aims to understand their potential roles in metabolic diseases. We categorized myokines into two groups based on regulation by aerobic and anaerobic exercise. With aging, the secretion of apelin, β-aminoisobutyric acid (BAIBA), bone morphogenetic protein 7 (BMP-7), decorin, insulin-like growth factor 1 (IGF-1), interleukin-15 (IL-15), irisin, stromal cell-derived factor 1 (SDF-1), sestrin, secreted protein acidic rich in cysteine (SPARC), and vascular endothelial growth factor A (VEGF-A) decreased, while that of IL-6 and myostatin increased. Aerobic exercise upregulates apelin, BAIBA, IL-15, IL-6, irisin, SDF-1, sestrin, SPARC, and VEGF-A expression, while anaerobic exercise upregulates BMP-7, decorin, IGF-1, IL-15, IL-6, irisin, and VEGF-A expression. Myostatin is downregulated by both aerobic and anaerobic exercise. This review provides a rationale for developing exercise programs or interventions that maintain a balance between aerobic and anaerobic exercise in the elderly.

## 1. Introduction

Owing to the improvements in healthcare and nutrition, life expectancy is increasing rapidly across the globe. It has been estimated that the elderly population, i.e., people over 60, will have increased by over 200% between 1970 and 2025 [1]. With the continued increase in life expectancy, health problems that afflict the elderly have garnered much attention. The prevalence of chronic diseases, such as diabetes, cancers, and cardiovascular, respiratory, and arthritic diseases, is increasing as the aging of the global population accelerates [2,3]. Skeletal muscle loss is among the most serious physical changes that the elderly experience. Approximately 75% of the individuals over 65 years of age do not indulge in the minimum physical activity required to stay healthy [4]. Skeletal muscle loss is a major health hazard as it is causally related to sarcopenia [5]. This muscular atrophy is associated with the development of sarcopenia, which is defined as a progressive decline in skeletal muscle mass and strength without loss of overall body weight that occurs with aging [6]. Individuals who spend much time sitting are prone to muscle mass loss and weakness, and therefore, they are at higher risk of developing sarcopenia [7]. In 1931, Critchley first recognized that the intrinsic mass of the hand and foot muscles decreased with aging [8]. Muscle weakness and decreased muscle mass negatively affect the elderly, both physiologically and psycho-socially, as they cause difficulty in performing daily tasks, increasing the risk of falling, reducing independence and quality of life, and increasing depression, social isolation, physical inactivity (sedentarism), susceptibility to chronic diseases, and rates of all-cause mortality [9,10,11]. The World Health Organization (WHO) announced that physical inactivity (5.5% of the total or 3.2 million) is the fourth most serious global health risk associated with mortality following high blood pressure, tobacco use, and high blood glucose [12].

Skeletal muscles are attached to bones by tendons and aponeuroses and are under the voluntary control of the somatic nervous system. They account for 30–50% of the total body mass and are primarily responsible for locomotion and metabolic homeostasis [13]. In recent decades, it has been discovered that contracting skeletal muscles release various hormone-like substances [14,15]. These activators are called myokines, which are small proteins (5–20 kDa) and proteoglycan peptides that are produced and secreted by skeletal muscle cells in response to muscle contractions [16,17]. Early in the study of myokines, it was hypothesized that high levels of exercise-induced circulating cytokine IL-6 released by immune cells (NK cells and CD8+ cells) were associated with muscle damage [18]. Although the plasma IL-6 concentration increases approximately 100-fold after exercise, it normalizes rapidly [19]. Electric stimulation inducing the rat gastrocnemius and soleus muscles produced contraction without injury and resulted in increased IL-6 [20]. Furthermore, in humans, the IL-6 turnover in the femoral arteries and veins has been demonstrated to be very sensitive to muscle exercise [21]. These results suggest that skeletal muscle contraction is associated with IL-6 secretion. However, it remains possible that free macrophages in the blood enter the skeletal muscle and secrete IL-6 during muscle contractions. Recently, with the development of molecular analysis techniques, such as quantitative real-time PCR and a new contractile C2C12 myotube model, it has been directly demonstrated that IL-6 is secreted by skeletal muscles [22,23,24,25]. Various myokines secreted by skeletal muscles during aerobic and anaerobic exercises have been studied in connection with various human diseases using state-of-the-art techniques, including total RNA sequencing [26,27,28]. For a long time, skeletal muscles were only recognized as being involved in the physical aspects of exercise. However, with the discovery of exercise-induced myokines, skeletal muscles have been demonstrated to be involved in the maintenance of metabolic homeostasis. Although the detailed mechanisms are not clear, both skeletal muscle contraction and mass maintenance appear to be actively involved in maintaining health and preventing disease development in the elderly, particularly considering the rapid deterioration of muscle physiology with aging.

Thus, studies on myokines are necessary for ensuring the maintenance of skeletal muscle health in the elderly. This review summarizes the myokines regulated by physical activity that are affected by aging and aims to understand their potential roles in metabolic diseases. We have reviewed 13 myokines whose secretion is influenced by aging but controlled by physical activity. We categorized these myokines into two groups, one regulated by aerobic exercise and the other by anaerobic exercise. With aging, the secretion of apelin, BAIBA, BMP-7, decorin, IGF-1, IL-15, irisin, SDF-1, sestrin, SPARC, and VEGF-A decreased, while that of IL-6 and myostatin increased. Aerobic exercise upregulates apelin, BAIBA, IL-15, IL-6, irisin, SDF-1, sestrin, SPARC, and VEGF-A expression, while anaerobic exercise upregulates BMP-7, decorin, IGF-1, IL-15, IL-6, irisin, and VEGF-A expression. Myostatin is downregulated by both aerobic and anaerobic exercise.

## 2. Myokines

### 2.1. Apelin

Human apelin was identified and isolated in 1998 as an endogenous ligand of the G-protein-coupled receptor APJ and was named the APJ endogenous ligand. The apelin gene located at chromosome Xq25–26.1, encodes a 77 amino acid preproprotein [29,30]. After the cleavage of the signal peptide, the protein is processed into various bioactive endogenous peptides, such as apelin-13, -16, -17, and -36 [31], which are widely expressed in various organs. Apelin regulates a wide range of physiological processes, including blood pressure [32], cardiac contractility [33], and angiogenesis [34], and is involved in pathophysiological processes underlying hypoxia [35], obesity [36], diabetes [37], and various cancers [38].

In one study, myokine expression in the vastus lateralis was examined in 11 obese nondiabetic males after cycling and running (aerobic exercise) for 8 weeks; in contrast to what is known for the other known myokines, this study reported a significant increase in the expression and secretion of apelin. This was the first study to recognize apelin as a myokine [39]. A reduction in apelin has also been observed in elderly subjects. In particular, abnormalities in muscle function grow more severe with age in apelin (-/-) mice. Thus, apelin has been suggested as a biomarker for diagnosing early sarcopenia and a target for the development of therapeutic agents for age-associated muscle weakness [40]. Also, Rai et al. showed that the downregulation of the apelinergic axis accelerated the onset and progression of aging [41].

The concentration of plasma apelin increases in response to aerobic exercise. Apelin mRNA expression increased by approximately 40% in hypertensive rats in response to swimming training for 9 weeks, and had positive effects on hypertension [42]. Apelin mRNA expression in the vastus lateralis was upregulated 3.3-fold after 11 obese males were subjected to aerobic exercise (cycling and running) for 8 weeks [39]. Skeletal muscle from mice subjected to aerobic exercise (treadmill and ladder) and C2C12 cells treated with the exercise mimetic, forskolin showed increased apelin plasma and mRNA concentrations, respectively [43]. Furthermore, a combination of apelin treatment and exercise (treadmill) significantly reduces ischemia-reperfusion injury in rats [44].

### 2.2. β-aminoisobutyric Acid (BAIBA)

BAIBA (C_4_H_9_NO_2_) is a small, non-protein myokine with a molecular weight of 103.6 Da that was first discovered in human urine in 1951 [45]. It is secreted by contracting muscles via the action of peroxisome proliferator-activated receptor-γ coactivator 1α (PGC-1α) and acts in a myokine-specific manner [46,47]. It is involved in various metabolic processes, such as acting on white adipose tissue to upregulate brown adipose tissue-specific genes, enhancing PGC-1α expression to increase lipid oxidation, suppressing inflammation in skeletal muscles, inhibiting cardiometabolic risk factors, and suppressing endoplasmic reticulum stress in hepatoblastoma cells. Blood BAIBA levels increase in response to continuous exercise [46,48,49,50]. The myokine properties of BAIBA suggest that many small molecule metabolites may have myokine functions.

Although its various functions are known, few studies have investigated the function of BAIBA in aging skeletal muscles. Plasma BAIBA levels are higher in young subjects than those in the elderly [51]. This could be because BAIBA expression is regulated by PGC-1α, and PGC-1α expression is lower in elderly subjects than that in young subjects [52,53]. This difference in PGC-1α expression is considered to be aging-dependent during acute exercise. BAIBA secretion is unchanged in young and old muscle; however, the expression of its receptor (Mas-related G-protein receptor Type D) decreases markedly in an age-dependent manner in osteocytes [54,55,56].

Secretion of BAIBA by skeletal muscles increases in mice overexpressing PGC-1α. Additionally, high plasma BAIBA concentrations are observed in humans undergoing wheel-running exercise but are inversely correlated with metabolic risk factors, which suggests that BAIBA may protect against metabolic diseases [46,57,58,59]. In one study, blood BAIBA levels were measured after 16 weeks of aerobic exercise training in Native American boys and girls (11–17 years) divided into obese and normal-weight groups. Individuals in the normal-weight group had 29% higher BAIBA levels than those in the obese group [60]. Physical inactivity in patients on hemodialysis reduces plasma BAIBA concentrations [61,62]. Kitase et al. reported that BAIBA functions as an osteocyte survival factor, protects against mitochondrial degradation due to ROS (reactive oxygen species), and regulates bone and skeletal muscle loss due to aging [54]. These results indicate that BAIBA is an important regulator of metabolic activity and is affected by a lack of exercise, which is frequently seen in the elderly.

### 2.3. Bone Morphogenetic Protein 7 (BMP-7)

In 1965, Urist recognized BMP as an important factor in osteogenesis and bone formation [63]. BMP-7, also called osteogenic protein-1, is a member of the TGF-β superfamily of cysteine-knot fold cytokine-growth factors [64]. Human *BMP-7* has been isolated and mapped to chromosome 20q13.31. This has been proposed as a possible locus for the Holt-Oram syndrome, which manifests as skeletal abnormalities of the upper limbs and hands [65,66,67]. BMP-7 is a multifunctional growth factor involved in cell proliferation, apoptosis, organ repair, and differentiation of brown adipose tissue, but its most important function is inducing cartilage and bone formation [68,69,70,71]. 

BMP-7 mRNA and protein levels are lower in elderly subjects, in both humans and rats, compared to young ones [72,73]. BMP-7 mRNA and protein are also expressed at low levels in articular cartilage of elderly subjects with osteoarthritis [74] owing to methylation of the BMP-7 promoter [75]. 

BMP-7 induces myotube formation in the mouse pluripotent mesenchymal precursor cell line C2C12 and causes increased expression of muscle differentiation factors such as BMP-1, GDF-6, and GDF-8 [76]. Winbanks et al. demonstrated that BMP-7 is a positive regulator of skeletal muscle mass in a neurogenic atrophy model [77]. Biomechanical stimulation (1 Hz for 3 h/day) increased BMP-7 mRNA expression in rat osteoblasts and inhibited posttraumatic osteoarthritis [78]. Expression of BMP-7 mRNA and protein increased in the gastrocnemius and lower limbs skeletal muscles of rats (rectus femoris, vastus lateralis, and gastrocnemius) after endurance training and gradual exercise, respectively [73,79].

### 2.4. Decorin

Human decorin is a small leucine-rich proteoglycan of 90–140 kDa that is associated with collagen fibrils in all connective tissue. The gene is located on chromosome 12q23 [80] and regulates transforming growth factor (TGF)-beta 1 activity as well as the cell cycle [81]. Decorin suppresses myostatin activity, which is associated with obesity and diabetes [47]. In 2014, decorin was first recognized as a myokine, and its levels in both plasma and skeletal muscle increase in response to physical activity [82].

A study that compared young (0–15 years) and old (16–83 years) individuals demonstrated that decorin mRNA expression decreases significantly in an age-dependent manner in human skin fibroblast [83]. In one study, the molecular weight of decorin isolated from the placenta of an old rat was estimated to be approximately 40 kDa, which is smaller than that of the normal protein (approximately 100 kDa). The smaller size was attributed to variations in the glycosaminoglycan base structure with aging [84]. Age-dependent changes in decorin have also been observed in human skin [85]. Although tendon injuries occur very frequently in the elderly, few studies have investigated the relationship between decorin and aging tendons. Dunkman et al. reported that decorin expression is closely related to the changes in aging tendons [86,87].

Articular cartilage decorin levels increase in beagle dogs after long-distance running exercise (40 km/day, 15 weeks) [88]. In 31 young men, decorin mRNA in the vastus lateralis, but not that in the patellar tendon, increased after exercise on a Krogh ergometer [89]. Anaerobic exercise upregulates decorin levels in the human plasma and *decorin* mRNA in the vastus lateralis, and regulates the expression of genes coding for proteins, such as follistatin, Myod1, atrogin1, and MuRF1, which are involved in hypertrophic pathways in the skeletal muscle [82]. When rats were subjected to moderate exercise on a treadmill, the expression of decorin at mRNA and protein levels increased, whereas they decreased in the calcaneal tendon during strenuous treadmill running. Increased decorin levels regulate matrix metalloproteinase-2 and tissue inhibitor of metalloproteinase-2 expression in the surrounding tissue, which makes the tendon stronger [90].

### 2.5. Insulin-Like Growth Factor 1 (IGF-1)

In 1957, IGF-1 was first recognized by Salmon and Daughaday as a “sulfation factor” that stimulates sulfate incorporation in rat cartilage [91]. In 1978, Rinderknecht and Humbel purified a human IGF-1, a protein of 70 amino acids with structural resemblance to proinsulin [92]. In 1983, the human IGF-1 cDNA was cloned, and in 1984, IGF-1 was found to be located on chromosome 12q23.2 [93,94]. Although IGF-1 is a multifunctional peptide, its main physiological function is as a growth hormone (GH) essential for normal bone and tissue growth and development [95]. In 2012, IGF-1 was recognized as a myokine produced and secreted by the muscle fibers [96].

Plasma IGF-1 decreases with age in humans, leading to GH release impairment, immunocompetence, and GH-IGF axis activity [97,98,99,100,101]. Furthermore, DNA synthesis and proliferation are observed in response to aging-associated low IGF-1 levels [102,103]. IGF-1 gene-ablated (IGF-1-/-) mice are characterized by severely impaired health [104] and poor mitochondrial function in the hippocampus [105]. IGF-1 levels have been reported to be low in community-dwelling elderly subjects and shows promise as a biomarker for sarcopenia [106,107].

Several studies have shown that impaired IGF-1 activity results in muscular dysfunction. Elderly marathon runners have high GH-IGF activity, which indicates that exercise in the elderly is closely related to IGF-1 activity [108]. IGF-1 is upregulated in response to anaerobic exercise, such as weightlifting in 72- to 98-year-old women [109]. Additionally, rats with spinal cord injury exhibited IGF-1 upregulation in the soleus after treadmill training, which resulted in the upregulation of muscle regeneration markers, such as myogenin and MyoD [110]. Significantly increased IGF-1 mRNA expression has been reported in postmenopausal women after a single-leg extension exercise [111].

### 2.6. Interleukin-15 (IL-15)

Human IL-15 was reported simultaneously by two groups in 1994 as a T-cell growth factor. *IL-15*, located on chromosome 4q31, encodes a 14–15 kDa glycoprotein incorporating a four α-helix bundle [112,113,114]. In humans, *IL-15* expression is detected in various cells and tissues, including skeletal muscle, epithelial cells, monocytes, and dendritic cells [115]. The primary biological functions of IL-15 are to activate and proliferate T-cells and NK cells, inhibit apoptosis, and accelerate CD8(+) antitumor immunity [116,117,118]. Brandt and Pedersen suggested in 2010 that muscle-derived IL-15 is a myokine constitutively expressed by skeletal muscles and is regulated in response to strength training [119].

The levels of plasma IL-15, quadriceps IL-15, and gastrocnemius IL-15 decline gradually with age in rodents [120,121]. Plasma IL-15 levels also decrease with age in humans [122]. Elderly subjects with sarcopenia have significantly lower plasma IL-15 levels, and studies have reported a relationship between sarcopenia and insufficient plasma IL-15 levels [123,124]. Age-dependent upregulation of IL-15 has been reported in splenic stromal cells but not in skeletal muscle [125].

Several studies have shown that exercise upregulates plasma IL-15 levels as well as *IL-15* mRNA and protein levels in skeletal muscle. In one study, a noticeable increase in plasma IL-15 levels was observed in 76 men and 77 women after 10 weeks of weight training (i.e., chest press, seated row, hamstring curl, incline press) [126]. Pérez-López et al. reported a significant increase in plasma IL-15 levels and muscle *IL-15* mRNA and protein levels after leg press and knee extension resistance exercises [127]. In rats with type 2 diabetes, the expression of IL-15 protein increased significantly in the soleus after 12 weeks of aerobic exercise (treadmill training) [128] and increased by 40% in the vastus lateralis of 30 men after 12 weeks of endurance exercise on a cycloergometer [129]. Upregulation of IL-15 after treadmill running increases fibroblast collagen synthesis and cell proliferation via the AMPK—Activated Protein Kinase, signaling pathway [122]. Hingorjo et al. demonstrated a twofold increase in plasma IL-15 levels after a single session of submaximal aerobic exercise [130]. Plasma IL-15 levels more than doubled in 37 athletes (11 females and 26 males) who performed acute long-distance (35 km) endurance exercise [131].

### 2.7. Interleukin-6 (IL-6)

IL-6 is a cytokine that plays multifunctional roles in the regulation of the immune system, nervous system, and glucose homeostasis [132,133]. IL-6 has several names, including interferon beta-2 (IFNB2), B-cell stimulatory factor 2 (BSF2), hepatocyte stimulatory factor, and hybridoma growth factor. Zilberstein et al. and Hirano et al. cloned full-length cDNAs encoding human IFNB2—a 23.7 kDa protein comprising 212 amino acids—and human BSF2—a novel interleukin comprising 184 amino acids—respectively [134,135]. Using in situ hybridization, Sutherland et al. identified that *IFNB2* is located on chromosome 7p15.3 [136]. The extramembrane (IL-6r) and intramembrane (gp130) domains of the IL-6 receptor were cloned in 1988 and 1990, respectively [137,138]. IL-6 is the first myokine produced and released into the supernatant when C2C12 myotubes and skeletal muscle fibers are induced to contract by electrical pulse stimulation [22,23].

Plasma IL-6 levels are relatively higher in elderly subjects compared to those in younger subjects, particularly in men [139]. Many studies have reported that plasma IL-6 levels increase with age [140,141,142,143]. Although the mechanism underlying age-related IL-6 upregulation is not fully understood, increases in IL-6 levels affect redox balance, mitochondrial physiology, and satellite cells in skeletal muscle [144,145,146]. Further studies are required to investigate sgp130 and sIL-6r expression, which is not always consistent with the increase in plasma IL-6 levels during aging.

IL-6 levels increase in urine, but not in plasma, after long-distance running [147]. Steensberg et al. demonstrated that plasma IL-6 levels increase with exercise, which contribute to the maintenance of glucose homeostasis [21]. Since then, many studies have reported increased IL-6 levels in response to exercise. IL-6 mRNA levels increased significantly after a single round of exercise in an in vitro exercise model employing human cultured vastus lateralis cells [148]. However, plasma IL-6 did not change in patients with progressive multiple sclerosis after acute endurance exercise (i.e., arm ergometry, rowing, and bicycle ergometry) [149]. These results indicate that the effects of exercise on IL-6 differ in healthy subjects and in diseased patients, which should be considered in future studies. No changes in baseline IL-6 levels, or those observed in response to different intensities of isovolumetric resistance exercise were observed in a study of elderly (≥65 years) men [150]. Furthermore, blood-flow-restricted resistance exercise does not regulate IL-6 expression [151]. In one study, IL-6 mRNA expression increased significantly in healthy men (20.5 ± 1.5 years) after high-intensity interval training [152]. In another study, patients with a spinal cord injury developed muscle atrophy and sympathetic nervous system dysfunction owing to the lack of an exercise-related IL-6 response [133]. Finally, IL-6 levels increased after acute strenuous exercise, i.e., a wheelchair marathon [153,154]. A recent study showed that plasma IL-6 levels are closely related to obesity, microglial function, and lactate production [155,156,157]. Pedersen et al. have published several studies on the relationship between exercise and IL-6 levels in great detail [19,158,159].

### 2.8. Irisin (Fibronectin Type III Domain Containing 5 [FNDC5]) 

Irisin, which is a novel myokine discovered in 2012, is expressed in a PGC-1α-dependent manner to produce FNDC5. This is followed by cleavage of the N-terminal signal peptide and C-terminal hydrophobic domain, resulting in the production of a 12 kDa glycoprotein that is secreted into the bloodstream and is involved in fat metabolism. FNDC5 is predominantly localized in the endoplasmic reticulum [160]. Genomic sequencing analyses indicate that *FNDC5* contains six exons and this gene has been mapped to chromosome 1p35.1 [161]. Although the irisin receptor is unknown, irisin is highly conserved in all mammalian species, which suggests highly conserved biological functions [162]. Recently, irisin has been hypothesized to be involved in the downregulation of insulin resistance pathway (ROS → p38 MAPK → PGC-1α → irisin → insulin resistance pathway), which is positively controlled by exercise and negatively controlled by aging [163].

Baseline circulating irisin levels are lower in older than in younger subjects [164]. When plasma irisin concentrations were measured in 715 Korean participants divided into three groups (40s, 50s, and 60s), the lowest concentrations were found in the oldest group [165]. Irisin mRNA expression is significantly lower in old rats with acute liver injury than that in young rats [166]. However, unlike plasma irisin, irisin in the cerebrospinal fluid of humans has been demonstrated to increase with age [167], and the shortening of telomere length with aging is negatively correlated with plasma irisin levels [168].

Aerobic exercise, such as running, swimming, and treadmill training, upregulates the expression of irisin at mRNA and protein levels, as well as irisin levels in the plasma, in humans, mice, and rats [169,170,171,172,173,174,175,176,177]. However, irisin production is higher in cardiac muscle than that in skeletal muscle, which suggests that skeletal muscle is probably not the main source of irisin. Therefore, it is necessary to accurately evaluate how much plasma irisin is secreted from the skeletal muscles [178]. Bubak et al. reported that temperature (33 °C or 7 °C) during exercise does not affect plasma irisin concentrations, and Fox et al. demonstrated that exercise increases plasma irisin concentrations [179,180]. Furthermore, the increase in irisin levels in response to exercise ameliorates the functional impairment of neurons, which suggests the possibility of treating Alzheimer’s disease [181,182,183].

### 2.9. Myostatin (Growth/Differentiation Factor-8 [GDF-8]) 

Myostatin (GDF-8), a member of the TGF-β superfamily, plays an important role in the negative regulation of skeletal muscle growth and is specifically expressed in developmental and adult skeletal muscle [184,185]. Myostatin, inhibited by follistatin, has recently attracted attention as a useful pharmacological target for preserving muscle mass and preventing atrophy [186]. McPherron et al. and Gonzalez-Cadavid et al. isolated and characterized the mouse myostatin and human myostatin genes, respectively [184,187]. In one study, plasma myostatin concentration in three groups of subjects (19–35, 60–75, and 76–92 years) were highest in the 76–92-year-old group, which suggests that plasma myostatin could be used as a biomarker for diagnosing age-associated sarcopenia [188]. 

Resistance training decreases plasma myostatin concentrations in healthy men, and acute resistance exercise decreases myostatin signaling by activating Notch signaling in rats [189,190]. Myostatin mRNA and plasma protein levels in rat decrease in response to aerobic exercise, and the same results have been reported in humans [191,192,193,194]. Patients with chronic heart failure show an increase in myostatin mRNA and protein; however, they decrease with exercise, indicating a beneficial anti-catabolic effect in patients with chronic heart failure [195,196]. The same result has been observed in patients with chronic kidney disease [197,198]. A combination of myostatin inhibition and endurance training is particularly effective for treating lipidomic abnormalities [199]. In some cases, different results have been reported depending on age, sex, exercise type (aerobic or anaerobic), and localization (plasma or muscle). The complex in vivo roles of myostatin have been studied and speculated upon [107,200,201,202,203], and it is an interesting target candidate for further research.

### 2.10. Stromal Cell-Derived Factor 1 (SDF-1) 

The expression of SDF-1—also called CXC motif chemokine ligand 12 (CXCL12), intercrine reduced in hepatomas (IRH), and pre-B cell growth-stimulating factor—is expressed in many cell types (i.e., fibroblasts, myoblasts, muscle fibers) [204]. This chemokine was originally described as a B-cell precursor stimulating growth factor secreted by a bone marrow stromal cell line [205]. CXCR4 and CXCR7 are the primary physiological receptors of SDF-1 [206,207], and the gene coding for this protein is located on chromosome 10q11.1 [208,209]. SDF-1-CXCR4 signaling occurs in the mesenchyme of limbs during early development and is directly responsible for the development of appropriately sized muscles [210], which indicates its important role in skeletal muscle regeneration [211,212].

Alzheimer’s disease (AD) is the most common form of dementia in the elderly. The Tg2576 mouse model of AD, in which the gene encoding human amyloid precursor protein is expressed, is an important AD animal model. CXCL12 levels are lower in AD patients compared to those in heathy controls, which suggests that CXCL12 is closely associated with aging [213]. The numbers of senescent fibroblasts increase in aged pigmented skin, where SDF-1 mRNA expression is reduced 3.55-fold [214]. SDF-1 expression is downregulated in the wounded skin of old mice, just as it is suppressed in an age-dependent manner in human skin [215]. Periyasamy-Thandavan et al. reported decreases in SDF-1 concentrations and bioactivity in the bone marrow interstitial fluid of older humans (18–40 vs. 60–85 years) and mice (3 vs. 22 months) [216]. *SDF-1* expression is negatively regulated by miR-141–3p in an age-dependent manner [216].

The concentration of plasma CXCL12 is enhanced in response to training on a bicycle ergometer [217], and rounds of acute exercise increase CXCL12 mRNA expression in the skeletal muscle [218]. Four weeks of treadmill running produced a 3-fold increase in CXCL12 mRNA and protein levels in the gastrocnemius of rats, which activated mTOR-p70S6K signaling to induce autophagy [219]. When C2C12 myotubes are mechanically stretched, CXCL12 production and release increase results in the activation of endothelial cell proliferation. CXCL12 KO (CXCL12-/-) mice suffer from severely impaired angiogenesis in skeletal muscles [220].

### 2.11. Sestrin

Sestrin was first discovered in 1994 as a target of the tumor suppressor *p53* and was referred to as p53-activated gene 26 (PA26). The gene coding for sestrin is located on chromosome 6q21 [221,222]. In mammalian cells, three different sestrin isoforms, which share high sequence homology, are encoded by genes located on different chromosomes, Sestrin1 on chromosome 6, Sestrin2 on chromosome 1, and Sestrin3 on chromosome 11 [223]. Sestrin acts as an intracellular leucine sensor to negatively regulate the target of rapamycin complex 1 (TORC-1) signaling by activating AMP-dependent protein kinase (AMPK), which prevents the development of sarcopenia and extends the life span [224]. Pathophysiological stressors, such as DNA damage and oxidative stress, upregulate sestrin expression, which negatively regulates aging by activating the AMPK/autophagy pathway and inhibiting the TORC1 signaling [225].

Sestrin2 is downregulated in the gastrocnemius muscle of old mice, which is characterized by a marginal reduction in sestrin2 mRNA and a significant reduction in sestrin2 protein [226]. The expression of cardiac sestrin2 also decreases with age [227]. In a study of 31 young (18–30 years) and 73 older (65–80 years) men without specific diseases in New Zealand and Australia, plasma sestrin1 and sestrin3 levels were found to decrease with age [228]. Rai et al. reported that concentrations of plasma sestrin1 and sestrin2 decrease significantly in elderly subjects [229]. An early study on *Drosophila* sestrin showed that inactivation of sestrin results in a significant increase in the development of age-associated pathologies, such as cancer, diabetes, muscle dystrophy, and chronic inflammation [230].

mRNA expression and plasma and skeletal muscle levels of sestrin1 and sestrin2 increase simultaneously after exercise [231]. Sestrin2 protein expression increases in response to aerobic exercise (i.e., swimming) in old mice [226]. Phosphorylation of sestrin2 in response to exercise induces p62 dephosphorylation, thereby inducing oxidative stress and selective autophagy [232]. Exercise-induced sestrin has been demonstrated to interact with AMPK, although the precise molecular mechanism remains unclear. Thus, sestrin is beneficial and may provide new insight into age-associated metabolic diseases [233,234,235].

### 2.12. Secreted Protein, Acidic, Rich in Cysteine (SPARC; Osteonectin/Basement-Membrane Protein 40)

In 1989, the human *SPARC* was first demonstrated to be located on chromosome 5q33.1 [236], and since then, it has been confirmed that the SPARC protein functions in a Ca^2+^-ion-dependent manner [237]. SPARC is a 43 kDa secretory matricellular glycoprotein that has multiple biological functions, including tumor-suppressing activity, cell differentiation, and cell adhesion in several organs and cell types [238,239,240]. SPARC was first recognized as a myokine in 2013 as a result of cell-stretching stimulation experiments on C2C12 myocytes [241].

In experiments on young (4–5 months) and old (>21 months) male rats, SPARC expression unchanged in skeletal muscle progenitor cells of the old rats, whereas SPARC protein expression decreased in the same rats [242]. Silencing of SPARC using siRNA led to a direct reduction in myofibril diameter. Therefore, age-related decreased expression of SPARC may induce sarcopenia development [243,244]. Other studies have reported that SPARC expression generally decreases with age (7–10 vs. 24–30 weeks) in the tibialis anterior of wild mice [245]. 

In 2013, it was first demonstrated experimentally that aerobic exercise (i.e., cycling) increases SPARC secretion twofold in human plasma, and the same result was obtained in C2C12 myoblasts stimulated with electric pulses [241]. Catoire et al. identified SPARC as a myokine whose expression is upregulated in response to acute exercise [246]. It has been suggested that SPARC is a tumor suppressor, as changes in SPARC expression affect sensitivity to radiation and chemotherapy in patients with colorectal cancer [247,248]. It has recently been reported that exercise-induced SPARC expression suppresses cancer, as azoxymethane (AOM)-induced colonic tumorigenesis is inhibited by exercise, but not in SPARC(-/-) mice [241]. However, plasma SPARC levels do not increase in response to a single brief supramaximal cycle sprint, which suggests that the SPARC in the plasma originates from organs other than skeletal muscle, so the origin of SPARC in the blood must be clarified [249]. However, SPARC has gained attention as a myokine because of its cancer-suppressing effects [250].

### 2.13. Vascular Endothelial Growth Factor A (VEGF-A)

VEGF-A, first discovered in 1983 [251], is encoded by a gene located on chromosome 6p21.1; the cDNA encoding VEGF-A was isolated in 1989 [252]. VEGF-A is a secreted, 46 kDa homodimer glycoprotein containing a highly conserved receptor-binding cysteine-knot structure. VEGF-A is one of the most important factors in the growth and survival of skeletal muscle in humans and animals [253].

We conducted a bibliographic search on whether physical exercise affects plasma VEGF-A concentration in the elderly; we referred to the 10 most relevant (of the 574) articles published between 1990 and 2013. Although four articles confirmed increases in plasma VEGF-A concentration after physical exercise, the other six articles found no changes [254]. The downregulation of VEGF-A mRNA and protein expression has been reported in aged human dermal microvascular endothelial cells [255] and in aged skeletal muscle obtained from the human vastus lateralis [256]. Experiments using VEGF-A KO (VEGF-/-) mice have revealed reduced gastrocnemius muscle mass (by 80–90%) [257]. 

However, various studies have shown that physical exercise upregulates VEGF-A. VEGF-A mRNA and protein levels increased twofold in skeletal muscle after one-legged knee extension training (anaerobic exercise) for 8 weeks in patients with heart failure [258]. The increase in reactive oxygen species (ROS) due to exercise increases the expression of PGC-1α, resulting in upregulation of VEGF-A expression in skeletal muscle [259]. In one study, when mice were made to run on wheels (aerobic exercise) for 14 days, VEGF-A expression increased by 34.6% in the skeletal plantaris, but VEGF-/- mice did not recover after exercise training [260].

## 3. Discussion

The most reasonable way to maintain healthy muscles is to increase muscle mass through appropriate physical exercise and leisure-time physical activity (LTPA), such as walking, dancing, gardening, hiking, and swimming. Many recent studies have proved that maintaining high-quality muscles through appropriate physical activity helps prolong life via various mechanisms [261,262].

Many studies have demonstrated the beneficial effects of physical activity on the physical health of the elderly as a solution for preventing aging-related diseases [263,264,265,266]. Generally, exercise burns fat, preventing the accumulation of fat in the body. This reduces the likelihood that controlling metabolic risk factors such as high triglyceride, low high-density lipoprotein (HDL) cholesterol level, and high blood pressure would result in reduced incidence of metabolic diseases to which the elderly are vulnerable. Many studies have demonstrated that physical activity prevents metabolic diseases and contributes to a prolonged life span [261,262]. Physical exercise enhances immunity and improves memory, which is beneficial in Alzheimer’s disease [267,268]. LTPA also decreases the risks of 13 types of cancer [269]. Maintaining the thigh circumference, a typical skeletal muscle, reduces the risks of developing heart disease and premature death [270]. Indeed, physically active people are at a lower risk of all-cause mortality than their physically inactive counterparts [271]. In line with previous research, many experimental studies have proved the positive impact of physical activity on cognitive ability in the elderly [272,273,274]. Overall, physical activity has a direct positive effect on maintaining the health of the elderly.

The biggest takeaway of our review is that both aerobic and anaerobic exercises exert positive effects on skeletal muscles by releasing various myokines that are beneficial to the elderly. Given that most studies on LTPA in the elderly have focused on aerobic exercises [275,276,277], it is worth broadening the scope of research by examining the need for anaerobic exercise. The incidence of typical muscle diseases in the elderly could be attributed to normal food intake without ATP production in the body, which results in an age-related decline in skeletal muscle mass and strength (sarcopenia). However, the exact cause and treatment are not fully understood. Therefore, further research should focus on myokines whose expression is exclusively induced by anaerobic exercise. Searching for the cause of skeletal muscle loss and its biomarkers will provide clues to the prevention of geriatric diseases and development of new treatments. This review provides scientific evidence that can be used by health practitioners to develop and promote exercise programs or interventions that maintain the balance between aerobic and anaerobic exercises for the elderly. Skeletal muscle loss in the elderly is not just a personal problem but also a social problem. Considering the huge medical costs associated with the treatment of sarcopenia and osteoporotic fractures [278,279]—which impose a huge tax burden on all members of society—recreational therapists, researchers, policymakers, and clinicians are urged to work together to improve the health of the elderly.

## 4. Conclusions

Myokines are secreted in response to skeletal muscle contractions during exercise and are promising anti-aging molecules due to their benefits in age-related diseases. Our review provides a conceptual basis for understanding different exercise-induced myokines and their therapeutic roles in helping the elderly remain healthy. We found that myokines are produced in response to regular exercise and play a key role in preventing or attenuating aging-related disease, such as dementia, obesity, diabetes, and cardiovascular and metabolic diseases. Although the 13 myokines reviewed are all stimulated by exercise, each has unique characteristics. In brief, apelin is an anti-aging factor and has positive effects on hypertension and ischemia-reperfusion injury when combined with exercise. BAIBA prevents metabolic diseases by acting as an osteocyte survival factor, protecting against mitochondrial breakdown, and attenuating bone and skeletal muscle loss. BMP-7 is an important factor in bone formation and skeletal muscle mass maintenance. Decorin, IGF-1, and SDF-1 have positive effects on tendon strength, bone and tissue development, and skeletal muscle regeneration, respectively. IL-15 facilitates fibroblast collagen synthesis and cell proliferation. IL-6 contributes to the maintenance of glucose homeostasis, obesity regulation, microglial function, and lactate production. Irisin might become a treatment for Alzheimer’s disease because of its positive influence on neuron functional impairment. The most interesting is myostatin. Unlike the other myokines, exercise reduces its secretion. It is beneficial in chronic heart failure, chronic kidney disease, and lipidomic abnormalities. Sestrin helps prevent the development of age-associated metabolic diseases and sarcopenia. SPARC, which is increased by aerobic exercise, has potential as a cancer treatment. VEGF-A, which is upregulated by both anaerobic and aerobic exercise, is involved in the growth and survival of skeletal muscle.
